# Prohibitin regulates the FSH signaling pathway in rat granulosa cell differentiation

**DOI:** 10.1530/JME-15-0278

**Published:** 2016-05-01

**Authors:** Indrajit Chowdhury, Kelwyn Thomas, Anthony Zeleznik, Winston E Thompson

**Affiliations:** 1 Department of Obstetrics and GynecologyMorehouse School of Medicine, Atlanta, Georgia, USA; 2 Reproductive Science Research ProgramMorehouse School of Medicine, Atlanta, Georgia, USA; 3 Department of NeurobiologyMorehouse School of Medicine, Atlanta, Georgia, USA; 4 Department of Cell Biology and PhysiologyUniversity of Pittsburgh, Pittsburgh, Pennsylvania, USA; 5 Department of PhysiologyMorehouse School of Medicine, Atlanta, Georgia, USA

**Keywords:** prohibitin, granulosa cells, differentiation, mitochondria

## Abstract

Published results from our laboratory identified prohibitin (PHB), a gene product expressed in granulosa cells (GCs) that progressively increases during follicle maturation. Our current *in vitro* studies demonstrate that follicle-stimulating hormone (FSH) stimulates Phb expression in rat primary GCs. The FSH-dependent expression of PHB was primarily localized within mitochondria, and positively correlates with the morphological changes in GCs organelles, and synthesis and secretions of estradiol (E_2_) and progesterone (P_4_). In order to confirm that PHB plays a regulatory role in rat GC differentiation, endogenous PHB-knockdown studies were carried out in undifferentiated GCs using adenoviral (Ad)-mediated RNA interference methodology. Knockdown of PHB in GCs resulted in the suppression of the key steroidogenic enzymes including steroidogenic acute regulatory protein (StAR), p450 cholesterol side-chain cleavage enzyme (p450scc), 3β-hydroxysteroid dehydrogenase (3β-HSD), and aromatase (*Cyp19a1*); and decreased E_2_ and P_4_ synthesis and secretions in the presence of FSH stimulation. Furthermore, these experimental studies also provided direct evidence that PHB within the mitochondrial fraction in GCs is phosphorylated at residues Y249, T258, and Y259 in response to FSH stimulation. The observed levels of phosphorylation of PHB at Y249, T258, and Y259 were significantly low in GCs in the absence of FSH stimulation. In addition, during GC differentiation FSH-induced expression of phospho-PHB (pPHB) requires the activation of MEK1-ERK1/2 signaling pathway. Taken together, these studies provide new evidence supporting FSH-dependent PHB/pPHB upregulation in GCs is required to sustain the differentiated state of GCs.

## Introduction

Mammalian granulosa cells (GCs) are multilayered somatic cells residing within the ovarian follicle that support oogenesis through proliferation, differentiation, and interactive communications. These events are under the control of endocrine factors, including gonadotrophins (follicle-stimulating hormone (FSH) and luteinizing hormone (LH)) (Havelock *et al.* 2004). During these processes, GCs produce and secrete multiple autocrine and paracrine factors, and steroid hormones that play important roles as regulators of oogenesis and folliculogenesis. The coordinated biosynthesis of steroids in the ovary is critical for progression of the reproductive cycle, successful ovulation, and eventually pregnancy (Jamnongjit & Hammes 2006). Gonadotrophin-induced synthesis and secretions of steroids, progesterone (P_4_) and estradiol (E_2_), involve multiple steroidogenic enzymes including steroidogenic acute regulatory protein (StAR), p450 cholesterol side-chain cleavage enzyme (p450scc), 3β-hydroxysteroid dehydrogenase (3β-HSD), and aromatase (*cyp19a1*) (Miller & Bose 2011). All steroidogenic pathways begin in the mitochondria, where regulation of the cellular steroidogenic capacity occurs through key enzymatic rate-limiting steps (notably p450scc and StAR) (Duarte *et al.* 2014, Prasad *et al.* 2015). Both p450scc and StAR are localized in a complex multicomponent ‘transduceosome’ on the outer mitochondrial membrane (OMM) and inner mitochondrial membrane (IMM) (Duarte *et al.* 2014, Prasad *et al.* 2015).

Prohibitin (PHB) is a member of the highly conserved ubiquitous protein family and plays a pleiotropic role in cell cycle control, differentiation, and senescence, in addition to having antiproliferative and anti-apoptotic roles (Chowdhury *et al.* 2016). A growing body of experimental evidence implicates PHB in the maintenance of integrity of mitochondrial structure, functions, inheritance, and cellular homeostasis (Thompson *et al.* 1999, 2001, 2003, Sutovsky *et al.* 2000, Kawashima *et al.* 2006, 2008, Artal-Sanz & Tavernarakis 2009, Chowdhury *et al.* 2016). Recently, PHB has been identified as a substrate for Ras-Raf and Akt, and is essential for activation of the Ras-Raf-MEK1-ERK1/2 signaling pathway (Rajalingam *et al.* 2005, Han *et al.* 2008). Previous studies have shown that PHB is widely expressed in rat ovary and its expression is regulated by age and follicular stage (Thompson *et al.* 1999, 2004). Moreover, recent studies in pre-antral GCs isolated from diethylstilbestrol (DES)-treated rats and antral GCs isolated from equine chorionic gonadotrophin (eCG)-treated rats have shown that PHB is regulated by FSH in a follicular stage-dependent manner *in vitro* and that the role PHB plays in regulating steroidogenesis is dependent on the differentiation status of GCs (Wang *et al.* 2013*a*,*b*). However, the specific roles and contribution of mitochondrial PHB and phospho-PHB (pPHB) in mediating differentiation of normal undifferentiated GCs (mainly steroidogenesis) is not known.

The experimental studies presented here were designed to decipher the physiological effects of knocking down endogenous PHB on steroid (E_2_ and P_4_) synthesis, secretions, and the activation of MEK1-ERK1/2 pathway in FSH-induced rat ovarian undifferentiated GC model. Immunoblot studies were carried out under these experimental conditions to analyze the expression levels of selective steroidogenic enzymes including StAR, p450scc, 3β-HSD, and *Cyp19a1*, which are commonly regulated in the steroidogenesis pathway at the protein level. Parallel studies were carried out to examine expression of the acidic (phosphorylated) isoform of PHB, phosphorylated-PHB (pPHB), and to decipher its relationship with activation of the MEK1-ERK1/2 pathway in FSH-treated GCs. In addition, we analyzed the role of PHB in FSH-induced mitochondrial morphological changes using assays that examined changes in the mitochondrial fusion protein optic atrophy 1 (OPA1). OPA1 is a nuclear-encoded large dynamin-like GTPase that is found in the mitochondrial intermembrane space and regulates both mitochondrial fusion and cristae morphogenesis during steroidogenesis (Wasilewski *et al.* 2012).

## Materials and methods

### Animals

Sprague–Dawley (SD) rats (female, 21 days old) were purchased from Charles River Laboratories. Animals were given food and water *ad libitum*, and kept under a regular day/night (12h light:12h darkness) cycle. All animal care handling procedures in this study were approved by the Institutional Animal Care and Use Committee in accordance with the guidelines of the National Institutes of Health (NIH) and the US Department of Agriculture, and approved by the Morehouse School of Medicine Animal Care and Use Committee.

### Primary GC cultures

Primary GC cultures were isolated from immature (23–25 days old) SD rat ovaries as described previously (Chowdhury *et al.* 2007). GCs from sexually immature 23- to 25-day-old rats are referred to as undifferentiated because they lack the presence of functional LH receptor and do not produce E_2_ or P_4_ under basal conditions, and these GCs have not been exposed to pubertal cyclic gonadotrophins. However, these cells respond to FSH with respect to the production of cAMP and induction of LH receptor activation of the E_2_ and P_4_ biosynthetic pathways (Bebia *et al.* 2001).

### Adenoviral infection of GCs and treatments

Undifferentiated GCs were grown on a 6-well culture dish (~2×10^6^cells/well; 2 ovaries/rat/plate) in M199 media supplemented with 10% fetal bovine serum (FBS). Subsequently, the medium was removed and cells were washed twice with M199 (antibiotics-free) and infected with or without adenoviral (Ad) vectors (Ad-eGFP-scrambled: adenovirus with scrambled sequence RNA with green fluorescent protein (GFP); Ad-eGFP-shPhb: adenovirus with siRNA designed for knockdown of PHB with GFP) at a multiplicity of infection (MOI) of 5, 10, and 20 plaque-forming units per cell (pfu/cell) with occasional rocking as described previously by Chowdhury *et al.* (2007, 2011, 2013). After 2h of incubation, the media was replaced with fresh M199 media without FBS and incubated for 24h. Infected GCs showed 95–100% GFP expressions. Twenty-four hours after exposure to adenoviruses, the media was replaced with fresh M199 media without FBS with testosterone (30ng/mL) in presence or absence of FSH (100ng/mL) for 48h.

For inhibitor studies, after 24h culture of GCs, the media was replaced with fresh M199 media without FBS in the presence or absence of MEK inhibitor (PD98059; PD 20μM), and DMSO as parallel control for 1h. Followed by MEK inhibitor pretreated GCs were treated with testosterone in presence or absence of FSH for 6h, since half-life of the MEK inhibitor is less than 8h. When reagents were dissolved in DMSO, the same concentrations of DMSO were added to the control cells medium. The final concentration of DMSO was less than 0.1%. The dose and time for inhibitor studies were based on Supplementary Table 1 (see section on supplementary data given at the end of this article) and previous publication (Chowdhury *et al.* 2013).

After completion of each experimental group, total proteins were isolated for further analysis by Western blot analysis as described below.

### Assessment of survival state of GCs after completion of treatments

A detailed analysis of the morphological changes occurring in GCs after 48h treatment with testosterone or FSH+testosterone was carried out using live cell photographs taken under an inverted epifluorescence microscope to image the green fluorescence signals for the eGFP-shPhb or the control eGFP-scrambled construct alone. Phase-contrast pictures were taken to assess the survival status. The percentage of apoptosis (Hoechst 33248 staining) and caspase-3 activity was determined as described previously (Chowdhury *et al.* 2007, 2011, 2013).

### Assessment of ultrastructural changes in GCs using transmission electron microscopy

Transmission electron microscopy (TEM) for GCs was carried out as described by Thompson *et al*. (1997). Mitochondrial and lipid droplets cross-sectional areas were determined using ImageJ software (NIH, Bethesda, MD, USA).

### Immunofluoresence microscopy

Immunofluorescence microscopy for GCs was carried out by methods previously described by Chowdhury *et al.* (2007).

### Isolation of S-100 fraction and mitochondria

S-100 (cytosolic) fractions and mitochondria were prepared as described previously (Chowdhury *et al.* 2007, 2011). Protein levels of cellular fractions were analyzed by Western blot analysis.

### Generation of PHB anti-phospho-Tyr^249^ and anti-phospho-Thr^258^/Tyr^259^ antibodies

PHB contains multiple residues that could serve as phosphorylation sites in FSH stimulation of GCs (Rajalingam *et al.* 2005, Han *et al.* 2008, Ande *et al.* 2009, Chiu *et al.* 2013). It has been reported that insulin phosphorylates PHB at Y114 and Y259 (Ande *et al.* 2009), whereas Akt phosphorylates PHB at T258 (Han *et al.* 2008). In addition, PHB phosphorylation sites also occur within the Raf1 binding domain of PHB (residues 243–272). The MEK1-ERK1/2-signaling pathway is known to be involved in GC survival (Moore *et al.* 2001, Dewi *et al.* 2002, Amsterdam *et al.* 2003, Su *et al.* 2006, Woods & Johnson 2007, Duarte *et al.* 2014, Chowdhury *et al.* 2016). Thus, based on our unpublished phosphosite bioinformatic studies using complete PHB amino acid sequences, we developed our own custom-made pPHB antibodies. The anti-phospho-Tyr^249^ (anti-pTyr^249^) and anti-phospho-Thr^258^/Tyr^259^ (anti-pThr^258^/Tyr^259^) rabbit phospho-specific antibody against PHB peptide Ac-EDIA[pY]QLSRSRN-amide phosphorylated at tyrosine 249 residue and Ac-LSRSRNI[pT][pY]LPAGQS-amide phosphorylated at threonine 258 and tyrosine 259 residues were custom prepared and affinity purified by Thermo Fisher Scientific. In brief, two rabbits were immunized with the PHB phosphopeptide. The terminal bleeds were adsorbed over a nonphosphopeptide column and then subjected to two successive purifications over a phosphopeptide column. ELISA analysis showed a greater than 1000-fold preference for phosphorylated peptide over nonphosphopeptide. The specificity of pPHB antibodies were analyzed by Thermo Fisher Scientific.

### Phosphatase treatment

Phosphatase treatment was carried out as described by Pon *et al.* (1986) and Thompson *et al*. (1997).

### Western blot analysis

GC protein extracts obtained from different treatment conditions were subjected to 1D or 2D gel electrophoresis and Western blot analysis, as described previously (Thompson *et al.* 2001, Chowdhury *et al.* 2007, 2011, 2013; given in Table 1). For 1D gel electrophoresis, equal amounts of protein (25μg) were applied to each lane. For 2D gel electrophoresis, 80μg of protein purified from mitochondrial fractions isolated from cultured GCs after treatment were focused in the first dimension immobilized pH gradient (IPG) 3–10 or 4–7 strips for 60kV-h using a Bio-Rad Protean IEF Cell and second dimension followed by the Western blot analysis. PHB antibody was used to detect protein spots corresponding to 30kDa marker.

**Table 1:** List of antibodies used for Western blot analysis.

**Primary antibody**	**Origin**	**Dilution used**
Prohibitin (PHB)	Rabbit polyclonal, Neomarks, Fremont, CA, USA	1:1000
Steroidogenic acute regulatory protein (StAR)	Rabbit polyclonal, Santa Cruz Biotechnology	1:1000
p450 cholesterol side-chain cleavage enzyme (P450scc)	Rabbit polyclonal, Santa Cruz Biotechnology	1:1000
Hydroxysteroid dehydrogenase-3β (3B-HSD)	Rabbit polyclonal, Santa Cruz Biotechnology	1:1000
Aromatase (*cyp19a1*)	Rabbit polyclonal, Abcam	1:1000
Total ERK1/2 and pERK1/2	Rabbit polyclonal, Cell Signaling	1:1000
Anti-OPA1	Rabbit polyclonal, Abcam	1:1000
Porin	Rabbit polyclonal, Cell Signaling	1:1000
Tubulin	Mouse monoclonal, Sigma-Aldrich	1:1000

### Quantification of the PHB phosphorylation and steroidogenic protein ratios

Quantitative analysis of the PHB phosphorylation and steroidogenic protein expression was carried out using a scanning densitometer and Multianalyst Software version 1.0.2 (Bio-Rad) as described previously (Chowdhury *et al.* 2013).

### Steroids secretion analysis

Spent medium from GC cultures was collected, centrifuged (900***g***, 5min) and kept at −80°C for hormone analysis. 17β-estradiol (E_2_) and progesterone (P_4_) concentrations in spent medium were measured using RIA (El-Hefnawy & Zeleznik 2001, Zeleznik *et al.* 2003, Parakh *et al.* 2006, Escamilla-Hernandez *et al.* 2008). The detection limitation of E_2_ was 0.05pg/mL, and the intra-assay and inter-assay coefficients of variation were 6 and 4%, respectively. The detection limitation of P_4_ was 0.31pg/mL, and the intra-assay and inter-assay coefficients of variation were 5 and 4%, respectively.

### Statistical analysis

All experimental data are expressed as mean±s.e.m. of three separate experiments, each carried out in replicate. Statistical analysis was carried out by one-way ANOVA using SPSS version 11.0. Multiple comparisons were done by Newman–Keuls test. Differences were considered to be significant at *P*≤0.05.

## Results

### Ultrastructural changes in GCs in response to FSH and testosterone

To delineate the role of PHB in GCs differentiation, we first examined the impact of gonadotrophin-induced steroidogenesis by using our established rat primary GC culture system *in vitro* (Supplementary Fig. 1).

**Figure 1:**
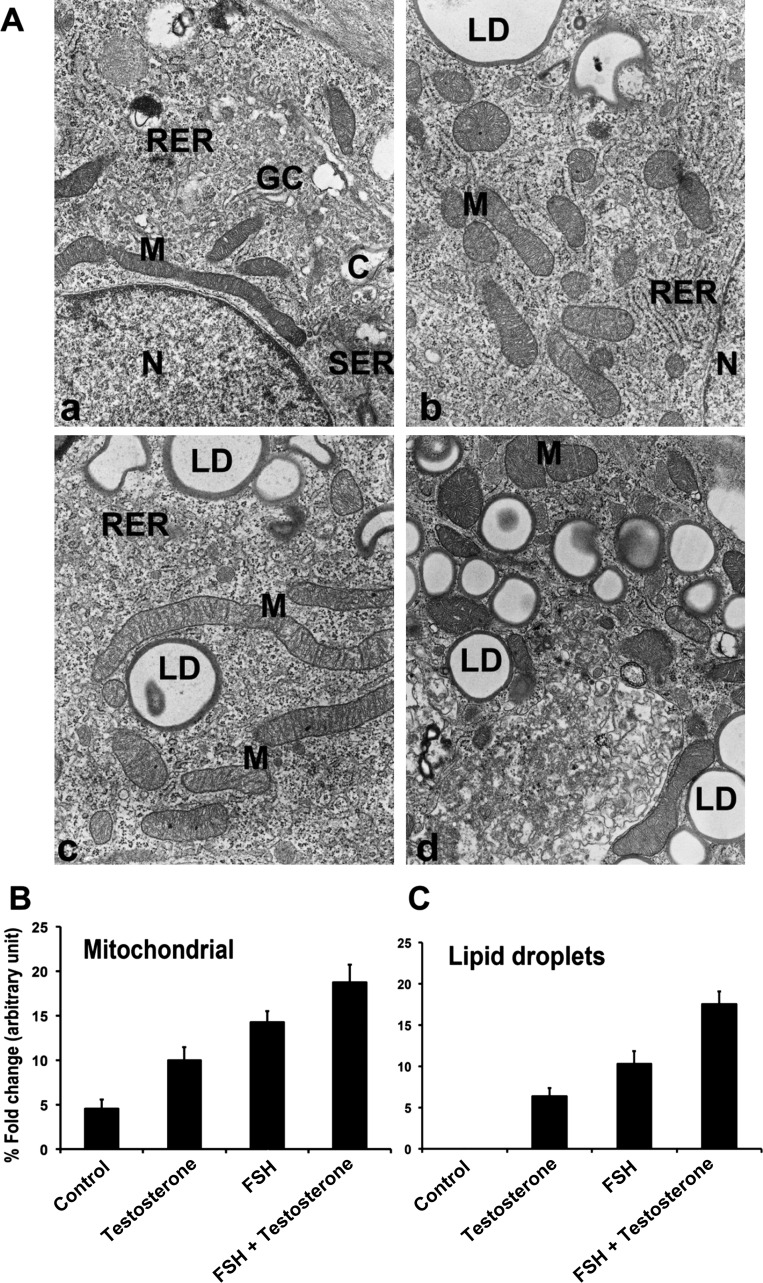
Ultrastructural changes in rat GC in response to FSH and testosterone. GCs were treated with or without FSH (100 ng/mL) and testosterone (30 ng/mL) or with FSH+testosterone for 48 h. Parallel control (C) GCs were maintained without any treatments. After completing the various treatments, GCs were fixed, mounted, and micrographs were taken with a TEM. (A) represents electron micrographs as control (a), testosterone (b), FSH (c), and testosterone+FSH (d). N, nucleus; M, mitochondria; GC, Golgi complex; RER, rough endoplasmic reticulum; SER, smooth endoplasmic reticulum; C, centriole; LD, lipid droplets. (B and C) are the cross-sectional areas of mitochondria and lipid droplets, respectively represented as percentage fold change (arbitrary unit). The bar graphs represent the mean±s.e.m. of results from three independent experiments (*n*=3). All groups are significantly different (*P*≤0.05).

TEM was used to analyze the effects of testosterone, FSH, or testosterone+FSH on mediating ultrastructural changes in undifferentiated and differentiated GCs. In this ultrastructure analysis, particular attention was paid to the oil droplet numbers and mitochondrial morphology of GCs under the respective culture condition (control, testosterone, FSH, and testosterone+FSH; Fig. 1A (parts a–d), B, and C). The cytoplasmic matrix of GCs cultured in control medium contained many ribosomes, no lipid droplets, few mitochondria with transversely oriented lamellar cristae, and rough endoplasmic reticulum (ER) (Fig. 1A (part a)). In GCs cultured with either testosterone or FSH alone, many cisternae of the rough ER and smooth ER with large number of elongated mitochondria were observed with few lipid droplets (Fig. 1A (b and c)). By contrast, GCs cultured with testosterone+FSH had many cisternae of the rough and smooth ER. It also had larger elongated mitochondria closely associated with some cisternae of the smooth ER with dense lipid droplets present (Fig. 1A (part d)) compared with GCs cultured with testosterone alone or FSH alone.

Based on results from Fig. 1 and Supplementary Fig. 1, testosterone with or without FSH was used in all subsequent studies that were carried out for the induction of steroidogenesis in GCs, where testosterone served as a substrate of aromatase (*cyp19a1*) as well as a paracrine regulator of FSH action (Hsueh *et al.* 1983, Thompson *et al.* 1997, El-Hefnawy & Zeleznik 2001, Zeleznik *et al.* 2003, Parakh *et al.* 2006, Wayne *et al.* 2007, Escamilla-Hernandez *et al.* 2008). In addition, in our subsequent studies, GCs were lysed and fractionated into mitochondrial and cytosolic fractions, and equal amounts of protein extracts were analyzed by 2D gel electrophoresis and Western blot analysis.

### Phb-knockdown inhibits steroidogenesis in gonadotrophin-induced GCs

As PHB is an important survival factor, knockdown of *Phb* sensitized the GCs (Chowdhury *et al.* 2016). Therefore, before delineating the effects of FSH in Phb-knockdown GCs, survival studies were carried out under these experimental conditions. The Supplementary Fig. 2 suggests that lower levels of knockdown of *Phb* do not affect the survivability of GCs. However, higher levels of knockdown of *Phb* in GCs tilt the balance of survivability toward cell death in a time-dependent manner.

**Figure 2:**
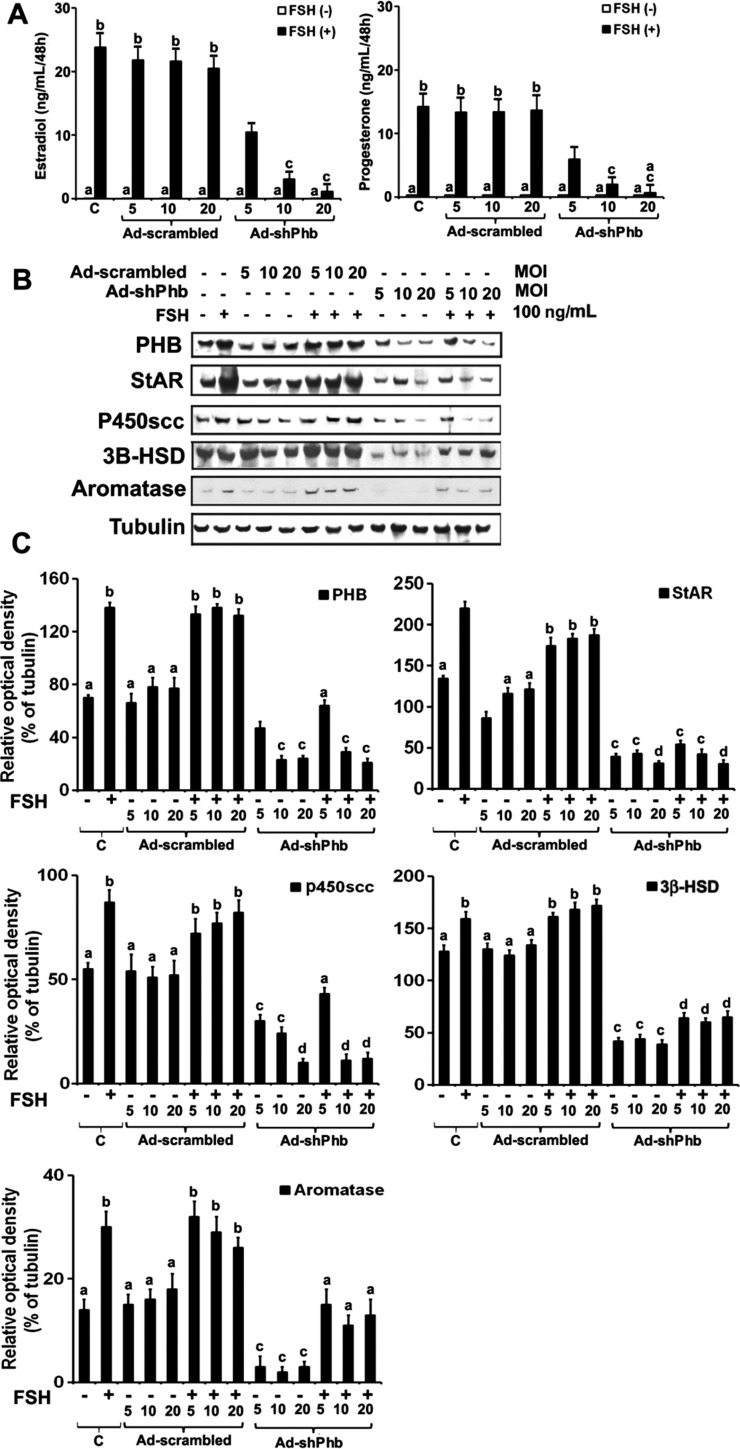
PHB knockdown inhibits steroidogenesis in gonadotrophin-induced rat GCs. GCs were transiently infected with Ad-shPhb (MOI 5, 10, and 20) or Ad-scrambled (MOI 5, 10, and 20) for 2 h and maintained in culture for 24 h in serum-free media and treated with testosterone (30 ng/mL) in presence or absence of FSH (100 ng/mL) for 48 h in serum-free media. (A) Bar diagram represents estradiol (E_2_) and progesterone (P_4_) secretions into the medium as measured by RIA. (B) Representative Western blot analyses of protein expression levels of PHB, StAR, p450scc, 3β-HSD, and aromatase in GCs induced by FSH under PHB knockdown experimental conditions. Equal amounts of protein (25 μg) were applied to each lane. Western blot analyses were analyzed for PHB, StAR, p450scc, 3β-HSD, and aromatase. Tubulin was used as an internal control for cytosol. (C) The bar graphs represent the relative percentage change in expression of PHB, StAR, p450scc, 3β-HSD, and aromatase as a ratio of the protein levels normalized by tubulin. The bar graphs represent the mean±s.e.m. of results from three independent experiments (*n*=3). All are significantly different (*P*≤0.05) except with same alphabets (a, b, c, and d). C, parallel control group.

To delineate the gonadotrophin effects on differentiation state after *Phb* knockdown in the GCs, we first examined whether infection with empty vector (Ad-scrambled) alone showed any changes in testosterone+FSH-induced P_4_ and E_2_ secretions in GCs. FSH+testosterone significantly stimulated P_4_ and E_2_ secretion (*P*<0.05; Newman–Keuls test) in Ad-scrambled-infected GCs (Fig. 2A). Similar results were observed in parallel controls without infected GCs treated with testosterone+FSH. Western blot analyses show (Fig. 2B and C) that testosterone+FSH significantly stimulated the expression of total PHB and key steroidogenic enzymes (StAR, p450scc, 3β-HSD, and aromatase (cyp19a1)) in Ad-scrambled infected and parallel controls (without infected) GCs treated with testosterone+FSH (*P*<0.05; Newman–Keuls test). These studies suggest that the Ad-scrambled construct has no adverse effect on the expression of PHB and steroidogenic enzymes, and P_4_ and E_2_ synthesis and secretions.

We then examined the effects of testosterone+FSH on secretions of P_4_ and E_2_ and key steroidogenic enzymes expression in the *Phb*-knockdown GCs. A significant dose-dependent knockdown of *Phb* expression occurred in Ad-shPhb-infected GCs treated with or without testosterone+FSH (*P*<0.05; Newman–Keuls test) (Fig. 2B). Interestingly, under dose-dependent knockdown of *Phb*, GCs when treated with testosterone+FSH showed a significant dose-dependent decrease in P_4_ and E_2_ secretions (Fig. 2A) with suppression of the key steroidogenic enzymes (StAR, p450scc, 3β-HSD, and aromatase) content (Fig. 2B and C) (*P*<0.05; Newman–Keuls test).

### Gonadotrophin stimulates phosphorylation of mitochondrial PHB in GCs

**Figure 3:**
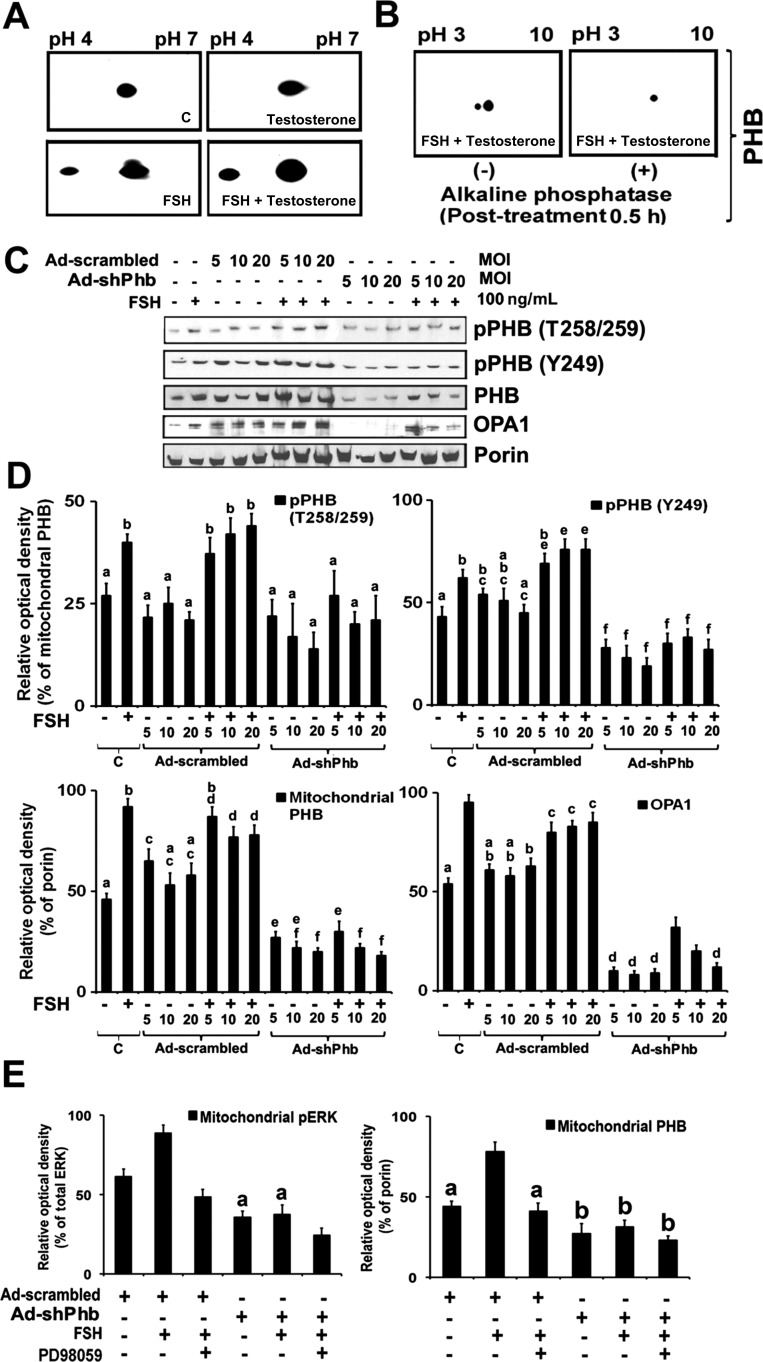
Gonadotrophin stimulates phosphorylation of mitochondrial PHB in GCs. (A) GCs were treated with or without FSH (100 ng/mL) and testosterone (30 ng/mL) or in combination (FSH+testosterone) for 48 h. Parallel controls (C) of GCs were maintained without any treatments. After 48 h, 80 μg of protein purified from the respective mitochondrial fractions were focused in the first dimension on IPG 4–7 strips for 60 kV-h using a Bio-Rad Protean IEF Cell and second dimension followed by the Western blot analysis. PHB antibody was used to detect protein spots corresponding to PHB. (B) Alkaline phosphatase-treated GCs. GCs were cultured in the presence of FSH+testosterone for 48 h. Mitochondrial fractions were collected and treated with and without alkaline phosphatase followed by separation on 2D gel electrophoresis, and PHB spots were identified by Western blot analysis. (C) Representative Western blot analyses of mitochondrial protein levels PHB, pPHB (T258/259 and Y249), and OPA1 in GCs induced by FSH under PHB silencing experimental conditions. After various treatments, mitochondrial fractions were isolated, and pPHB and OPA1 were analyzed by Western blot analyses. Equal amounts of protein (25μg) were applied to each lane, and Western blot analyses were analyzed for PHB, pPHB, and OPA1. Porin was used as an internal control for mitochondria. (D) The bar graphs represent the relative percentage change in expression levels of PHB, pPHB, and OPA1 as a ratio of the protein levels normalized by porin from three independent experiments as mean±s.e.m. All are significantly different (*P≤*0.05) except with same alphabets (a, b, c, d, e, and f). (E) The bar graphs represent the relative percentage change in expression of mitochondrial PHB/porin and pERK/ERK in GCs induced by FSH under PHB silencing experimental conditions. After various treatments, mitochondrial protein was isolated and analyzed by Western blot analyses for PHB, pERK, and ERK. Porin was used as an internal control for mitochondria. The bar graphs represent the mean±s.e.m. of results from three independent experiments (*n*=3). All are significantly different (*P*≤0.05) except with same alphabets (a and b).

Co-localization studies shown in Supplementary Fig. 1D suggest that FSH+testosterone-dependent upregulated PHB is mainly localized to mitochondria, and confirmed that the mitochondria is the key organelle for steroidogenesis (Poderoso *et al.* 2008, Miller & Bose 2011, Wasilewski *et al.* 2012, Duarte *et al.* 2014, Prasad *et al.* 2015). Moreover, previous studies have shown that PHB is a substrate for MEK-ERK pathway (Rajalingam *et al.* 2005, Han *et al.* 2008, Chowdhury *et al.* 2013, 2016). Therefore, we designed further studies that aimed at (i) determining whether mitochondrial PHB is phosphorylated (pPHB) during either FSH or testosterone or testosterone+FSH treatments and (ii) determining whether MEK-ERK signaling pathway was involved in FSH-induced mitochondrial PHB expression in GCs. After completion of each treatment, mitochondrial protein fractions were isolated and 2D Western blot analysis was carried out to analyze for PHB expression. As shown in Fig. 3A, PHB within the mitochondrial fraction in the controls (without treatment) and testosterone-treated GCs both have single spots, whereas a shift in the mobility of the PHB protein was observed toward the acidic region in 2D Western blot analysis with two distinct spots seen in the FSH and testosterone+FSH-treated groups. The level of PHB increased when GCs were cultured in the presence of FSH alone and FSH+testosterone, and the GCs were actively producing P_4_ and E_2_ (Fig. 2A). These results suggest that under these experimental conditions, the PHB protein has an acidic isoform that is most likely due to phosphorylation. To further determine whether these results are correct, mitochondrial pPHB in testosterone+FSH-treated GCs protein fractions were purified from isolated mitochondria and incubated with alkaline phosphatase. This phosphatase treatment resulted in disappearance of the acidic mitochondrial isoform appearing at isoelectric point 5.6 (Fig. 3B). These experimental results suggest that PHB within the mitochondrial fraction is phosphorylated with stimulation of FSH+testosterone.

We further explored whether mitochondrial pPHB and OPA1 expression were affected by testosterone+FSH treatment after knockdown of *Phb* expression in the GCs. We found that mitochondrial PHB was phosphorylated at residues Y249, T258, and Y259 in response to FSH+testosterone stimulation (Fig. 3C). In addition, the levels of phosphorylation of PHB at Y249, T258, and Y259 were significantly higher in FSH+testosterone treated GCs than was observed in the parallel controls GCs (*P*<0.05; Newman–Keuls test). Interestingly, the levels of PHB phosphorylation at Y249 and T258/Y259 were significantly low in GCs without FSH treatment (*P*<0.05; Newman–Keuls test). Moreover, there was a significant increase in mitochondrial pPHB (Y249 and T258/Y259), PHB, and OPA1 expression in Ad-scrambled-infected GCs treated with testosterone+FSH, which is similar to that observed in control GCs (uninfected Ad-virus) treated with FSH+testosterone (*P*<0.05; Newman–Keuls test; Fig. 3C and D). By contrast, a significant dose-dependent knockdown of mitochondrial *Phb* expression occurred in Ad-shPhb-infected GCs with a concomitant decrease in OPA1 expression (*P*<0.05; Newman–Keuls test; Fig. 3C and D). Furthermore, Ad-shPhb-infected GCs treated with testosterone+FSH showed significantly (*P*<0.05; Newman–Keuls test) lower concentrations of pPHB (Y249 and T258/Y259) and OPA1 compared with Ad-scrambled-infected GCs treated with testosterone+FSH.

We further examined whether FSH stimulates MEK-ERK pathway in *Phb* knockdown GCs alone or in combination with MEK inhibitor (PD98059). As shown in Fig. 3E, under FSH stimulation ERK1/2 is phosphorylated at significantly higher levels compared with the parallel control groups (*P*<0.05; Newman–Keuls test). Interestingly, *Phb* knockdown in GCs with FSH stimulation resulted in a marked decrease in the levels of ERK1/2 phosphorylation compared with the parallel control groups (*P*<0.05; Newman–Keuls test). Moreover, the presence of MEK inhibitor PD98059 with stimulation by FSH+testosterone resulted in drastically inhibited ERK1/2 phosphorylation (Fig. 3E). Consistently, we observed that depletion of PHB had a negative impact on FSH-induced phosphorylation of ERK1/2 without changes in its total protein expression levels.

## Discussion

This study demonstrates for the first time that PHB/pPHB plays a key differentiation role in activating the steroidogenic pathway and regulating the expression of the steroidogenic proteins in gonadotrophin-induced differentiation model of rat ovarian undifferentiated GCs. In our experimental culture system, we have shown that GCs have a strong steroidogenic response to FSH+testosterone, which is in agreement with several published observations that FSH+testosterone stimulates StAR, 3β-HSD, p450scc, and aromatase (*Cyp19a1*) expression, and E_2_ and P_4_ secretions (Hsueh *et al.* 1983, El-Hefnawy & Zeleznik 2001, Eimerl & Orly 2002, Zeleznik *et al.* 2003, Parakh *et al.* 2006, Wayne *et al.* 2007, Escamilla-Hernandez *et al.* 2008). Moreover, the TEM studies showed that the steroidogenic activity of GCs is supported morphologically by the appearance of smooth and rough ER, an apparent increase in the size and number of the mitochondria, and an increase in the number of oil droplets present in both the FSH or FSH+testosterone-treated GCs when compared with untreated parallel control GC (Thompson *et al.* 1997). Both mitochondria and ER play a critical role in steroidogenesis (Walther & Farese 2009, Miller & Bose 2011). Under these experimental conditions, the expression level of PHB is upregulated in presence of testosterone, FSH, and testosterone+FSH, suggesting that PHB plays a role in the steroidogenic pathway in undifferentiated GCs (Thompson *et al.* 2004). Therefore, in subsequent studies, dose-dependent knockdown of Phb was carried out in GCs using Ad-shPhb construct followed by treatment with FSH. The decrease in E_2_ and P_4_ secretion occurring in Phb-knockdown GCs treated with FSH is due to loss of StAR, 3β-HSD, p450scc, and aromatase (*Cyp19a1*) expression, which are known to govern steroidogenic pathways (Miller & Bose 2011, Duarte *et al.* 2014). The StAR is the acute steroidogenic response to tropic stimuli and facilitates cholesterol transport from the OMM to the IMM for steroidogenesis (Pescador *et al.* 1997, Wayne *et al.* 2007, Miller & Auchus 2011).

Our studies have also confirmed that phosphorylation is a fundamental post-translational modification that regulates PHB function, localization, and binding specificity of the protein (Rajalingam *et al.* 2005, Han *et al.* 2008, Ande *et al.* 2009, Chowdhury *et al.* 2016). Interestingly, all functional phosphorylation sites (Y249 and T258/Y259) of PHB are within the Raf1 binding region of PHB residues (residues 243–272) (Rajalingam & Rudel 2005, Chiu *et al.* 2013). However, whether there are direct interactions between these PHB phosphorylation sites with Raf have not been clarified in the current studies. The functional role that PHB/pPHB in the mitochondria plays in GCs differentiation may be explained by altered processing of OPA1 (Merkwirth *et al.* 2008). The reduced PHB/pPHB content in mitochondria resulting from Phb-knockdown GCs also resulted in inhibition of OPA1 expression in the mitochondria and is likely to be one of the mechanisms that explains the role of PHB in stabilizing the mitochondrial integrity and membrane potential and its subsequent involvement in regulating steroidogenesis in mitochondria (Artal-Sanz *et al.* 2003, Chowdhury & Bhat 2010, Chowdhury *et al.* 2016). Previous studies have strongly supported our results that OPA1 is absolutely required for maintaining mitochondrial morphology and ultrastructure for efficient support of steroidogenesis (Wasilewski *et al.* 2012). The detailed mechanisms by which PHB/pPHB affects OPA1 processing are currently unknown.

In addition, our current studies have demonstrated a role for PHB in MEK-ERK signaling pathway during gonadotrophin-stimulated steroidogenesis in GCs. The role for Ras-Raf-MEK-ERK signaling in regulating gonadotrophin-induced steroidogenesis in mammalian and hen GCs has been well studied (Bygrave & Roberts 1995, Moore *et al.* 2001, Richards 2001, Baines *et al.* 2002, Dewi *et al.* 2002, Cottom *et al.* 2003, Alonso *et al.* 2004, Hunzicker-Dunn & Maizels 2006, Su *et al.* 2006, Woods & Johnson 2007, Wayne *et al.* 2007, Duarte *et al.* 2014). Based on our studies, it appears that pPHB/PHB in the GC plays an indispensable role in MEK1-ERK1/2 activation. Furthermore, we have also observed that a decrease in PHB levels in the GCs inhibited MEK1-ERK1/2-signaling cascades and resulted in inhibition of GCs differentiation. Moreover, mitochondrial fusion is associated with the efficient localization of StAR and ERK activity in mitochondria (Poderoso *et al.* 2008, Duarte *et al.* 2014). Our data in this study further suggests that PHB is required for the phosphorylation of ERK in FSH-stimulated GCs.

Upon induction of steroidogenesis by FSH, mito­chondrial PHB through pPHB mediates the activation of pMEK-pERK expression that subsequently enhances steroidogenic pathway activity and stimulates E_2_ and P_4_ synthesis and secretions. An alternative mechanism that can explain the inhibition of steroidogenesis after mitochondrial *Phb* knockdown is by inhibition of steroidogenic enzymes in the mitochondria, thus protecting the mitochondria from undergoing functional incapacitation. Of note, however, our *in vitro* experimental results were not in agreement with previously published work that gonadotrophin treatment had an inhibitory effect on the mRNA/protein expression levels of PHB in GCs (Wang *et al.* 2013*a*,*b*). This apparent discrepancy could be explained by differences in the gonadotrophin dosages that were used in the respective studies, and the fact that the isolated DES and eCG primed pre-antral and antral GCs were then further treated with gonadotrophin *in vitro* (Boone *et al.* 1995, Karakji & Tsang 1995*a*,*b*, Minegishi *et al.* 1997, Simoni *et al.* 1997, Pellatt *et al.* 2011, Scheetz *et al.* 2012).

In summary, this study indicates that gonadotroph­in-dependent steroidogenesis in undifferentiated GCs are influenced not only by the expression of the key steroidogenic enzymes, but also by the 30-kDa PHB/pPHB, which acts as an obligatory mitochondrial protein that regulates mitochondrial function and, thus plays a central role as a key modulator of GC differentiation.

## Supplementary data

This is linked to the online version of the paper at http://dx.doi.org/10.1530/JME-15-0278.

## Declaration of interest

The authors declare that there is no conflict of interest that could be perceived as prejudicing the impartiality of the research reported.

## Funding

This study was supported in part by NIH Grants 1RO1HD057235, HD41749, 1SC3 GM113751, and G12-RR03034. This investigation was conducted in a facility constructed with support from Research Facilities Improvement Grant #bib6 RR18386 from NIH/National Center for Research Resources (NCRR). Part of this work was presented at the 43rd Annual Meeting of the Society for the Study of Reproduction in Milwaukee, WI, USA, 30 July–3 August 2010; the 46th Annual Meeting of the Society for the Study of Reproduction in Montreal, Canada, 22–26 July 2013; the 47th Annual Meeting of the Society for the Study of Reproduction, Grand Rapids, MI, USA, 19–23 July 2014; and 14th Research Centers in Minority Institutions (RCMI) International Symposium (Women’s Health), Washington, DC, USA, 1–3 December 2014.
